# Linkable Ring Signature for Privacy Protection in Blockchain-Enabled IIoT

**DOI:** 10.3390/s25123684

**Published:** 2025-06-12

**Authors:** Fang Guo, Yulong Gao, Jian Jiang, Xueting Chen, Xiubo Chen, Zhengtao Jiang

**Affiliations:** 1 State Key Laboratory of Media Convergence and Communication, Communication University of China, Beijing 100024, China; 202312063026@mails.cuc.edu.cn (F.G.); cenchen@cuc.edu.cn (X.C.); z.t.jiang@163.com (Z.J.); 2 State Key Laboratory of Networking and Switching Technology, Beijing University of Posts and Telecommunications, Beijing 100876, China; flyover100@163.com

**Keywords:** blockchain, privacy protection, post-quantum cybersecurity, information security, ring signature

## Abstract

The blockchain-enabled industrial Internet of Things (IIoT) faces security threats such as quantum computing attacks and privacy disclosure. Targeting these issues, in this study, we design a new lattice-based linkable ring signature (LRS) scheme, which is used to achieve privacy protection for the blockchain-enabled IIoT. Firstly, by using the trapdoor generation algorithm on the lattice and the rejection sampling lemma, we propose a new lattice-based LRS scheme with anti-quantum security and anonymity. Then, we introduce it into blockchain. Through the stealth address and key image technologies, we construct a privacy protection scheme for blockchain in the IIoT, and this LRS scheme protects identity privacy for users through anonymous blockchain. In addition, it also can resist the double spending attack with the linking user’s signature. Lastly, we provide a security analysis, and it is proven that our ring signature scheme satisfies correctness, anonymity, unforgeability and linkability. Compared with other similar schemes, the performance simulation indicates that our scheme’s public key and signature are shorter in size, and its computation overhead and time cost are lower. Consequently, our novel LRS scheme is more secure and practical, which provides privacy protection and anti-quantum security for the blockchain-enabled IIoT.

## 1. Introduction

Recently, the integration of blockchain and the industrial Internet of Things (IIoT) has emerged as a novel trend and area of research within industrial applications [1,2]. However, smart objects attached to the IIoT interact with humans and also process their private data [3,4]. Moreover, information is collected in large quantities and disclosed to the Internet without the consent of specific persons. In data storage, privacy disclosure stands out as one of the most serious threats within the IIoT [5,6].

In 2008, Nakamoto proposed blockchain for the first time [7]. However, with the in-depth study of blockchain, more and more people take a skeptical attitude towards blockchain’s anonymity in bitcoin trading [8,9,10,11]. In 2016, Zerocash was designed, which satisfies anonymity [12]. Saberhagen et al. designed the CryptoNote protocol [13]. Afterwards, Monero was proposed based on this CryptoNote protocol by using a ring signature to achieve anonymity. Thus, the ring signature was proposed [14]. Then, Liu et al. proposed a new kind of ring signature scheme, which was called the linkable ring signature (LRS) [15]. In LRS, not only does the signer’s identity in a ring signature remain anonymous but two ring signatures that are signed by the same signer can also be linked [16]. Considering its advantages, the LRS scheme is suitable in many different practical applications, such as e-voting, e-money, supply chains, and healthcare [17,18,19]. However, it still faces a problem, which cannot be ignored in Monero and Zerocash [20]. More specifically, in 2018, Fedorov clearly pointed out the security risks of quantum computing in blockchain [21].

Fortunately, lattice-based cryptography has been proven to have anti-quantum security [22]. Afterwards, Ajtai provided an innovative algorithm for constructing random short lattices [23,24]. Gentry et al. put forward a trapdoor function to construct a cryptographic algorithm [25]. Subsequently, some lattice-based signature schemes were proposed based on the bonsai tree algorithm [26]. In 2010, Agrawal et al. proposed an efficient lattice basis delegation algorithm [27]. By using this algorithm, Wang proposed an identity-based ring signature scheme [28]. Lyubashevsky provided the rejection sampling lemma for constructing lattice-based signatures without using the hash-and-sign methodology. This signature scheme is provably secure based on the worst-case hardness of the $\tilde{O}\left(n^{1.5}\right)$-SIVP problem [29]. Then, a new ring signature scheme on lattice was proposed, and its security was proven under the random oracle model [30].

Facing quantum computing attacks and privacy disclosure issues in blockchain-enabled IIoT, we propose a new LRS scheme and introduce it into blockchain to design a new post-quantum blockchain (PQB) with privacy protection for IIoT.

The rest of this study is organized as follows. In Section 2, some definitions and lemmas of the lattice theories are presented. In Section 3, we propose a secure LRS scheme based on lattice. A security proof is presented in Section 4. In Section 5, this LRS scheme is introduced into blockchain; we construct a privacy protection scheme for blockchain in IIoT and present a performance analysis and efficiency comparison of our scheme with other schemes. In Section 6, conclusions are provided.

## 2. Preliminaries

### 2.1. Lattice and Hard Problem

In the following, R denotes the real number set, and Z denotes the positive integer set. Therefore, we use $R^{m}$ to stand for the m-dimensional Euclidean vector space. In general, m∈Z,n∈Z,m≥n. L and Λ  denote lattice in the following content, vector $x=(x_{1},x_{2},\cdots ,x_{n-1},x_{n}{)}^{T}$ in the space $Z^{m}$, and $\left\|x\right\|=\sqrt{x_{1}^{2}+x_{2}^{2}+\cdots x_{n-1}^{2}+x_{n}^{2}}$.

**Definition 1.**  (Lattice): Suppose that there are *n* linearly independent vectors $v_{1},v_{2},\cdots ,v_{n}\in Z^{m}$, so lattice *L* can be represented as follows.

(1) $$L(v_{1},v_{2},\cdots ,v_{n})=\left\{\sum_{i=1}^{n}a_{i}v_{i}\left|a_{i}\in Z,i=1,\cdots ,n\right.\right\}$$

**Definition 2.**  (Lattice shortest independent vector problem (SIVP)): Given an *n*-dimensional lattice L, output a set of *n* linearly independent vectors $s_{i}$ and $\left\|s_{i}\right\|\leq \lambda_{n}\left(L\right)\;(i=1,\ldots ,n).$

**Definition 3.**  (Lattice SIS problem): Suppose that we have a real constant v>0 and a matrix $A\in Z_{q}^{n\times m}$ where *q* is a prime. Find a non-zero vector $x\in Z^{m}$ such that Ax≡0mod⁡q  and $\left\|x\right\|\leq v$.

### 2.2. Trapdoor and Lemmas

**Lemma 1**  [25]. Select a matrix $A\in Z_{q}^{n\times m}$ where prime number *q* > 2, B is a basis of $\Lambda_{q}^{⊥}(A)$, the Gaussian parameter $s\geq ||\tilde{B}||\omega (logm)$. Thus, for any vector $y\in Z_{q}^{n}$, there exists a probabilistic polynomial time (PPT) algorithm SamplePre(A,B,y,s), which can output a vector $e\in Z_{q}^{m}$ from a distribution that is close to $D_{\Lambda_{q}^{⊥}(A),s}$.

**Lemma 2**  [25]. For any prime q=poly(n) and any $m\geq 2n\log^{2}q$, there exists a PPT algorithm $TrapGen(1^{n})$, which can output a matrix $A\in Z_{q}^{n\times m}$ and a full-rank basis $S\in Z^{m\times m}$ of $\Lambda^{⊥}(A)$. And the distribution of **A** is statistically close to uniform over $Z_{q}^{n\times m}$, such that AS=0(mod⁡q) and $\left\|S\right\|=O(m^{0.5})$.

**Lemma 3**  [27]. For a matrix $A\in Z_{q}^{n\times m}$, the *m*-dimensional lattice $\Lambda_{q}^{⊥}(A)$, the Gaussian parameter $s\geq \left\|\tilde{T}\right\|\cdot s_{R}\sqrt{m}\omega (\sqrt{\log^{3/2}m})$, input a basis **T** of the lattice $\Lambda_{q}^{⊥}(A)$, and it has the nonsingular matrix $R=T^{-1}$, $R\in Z^{m\times m}$. By running the PPT algorithm BasisDel(A,R,T,s), a basis $B\in Z^{m\times m}$ of $\Lambda^{⊥}(AR^{-1})$ can be obtained and with overwhelming probability $\left\|\tilde{B}\right\|<s/\omega (\sqrt{\log m})$.

**Lemma 4. Rejection sampling**  [29]. Suppose that *V* is a subset $Z^{m}$ and each element *v* has norm less than *t*, $\sigma =\omega (t\sqrt{\log m})$, and h:V→R is a probability distribution. Thus, there is a constant M=O(1) such that output distribution statistical distance of the following two algorithms is less than $2^{-\omega (\log m)}/M$.

Algorithm A: v←h, $x\leftarrow D_{v,\sigma }^{m}$, output (*x*,*v*) with the probability $\min(D_{\sigma }^{m}(x)/MD_{v,\sigma }^{m}(x),\;1)$.

Algorithm B: v←h, $x\leftarrow D_{\sigma }^{m}$, output (*x*,*v*) with the probability 1/M.

The probability that algorithm A outputs something is at least $(1-2^{-\omega (\log m)})/M$.

### 2.3. Related Work

Some related works from recent years of the blockchain-based LRS schemes and privacy-preserving blockchain mechanisms for IIoT are given in this section. In 2018, Wang et al. proposed a lattice-based ring signature scheme without trapdoors [31]. Gao et al. presented a new ring signature scheme from lattice in 2019 [32]. Through experiments under the same parameters, we find that this scheme’s signature size is large, which leads to its low efficiency. In 2021, Le et al. presented an identity-based LRS (IdLRS) in both integer lattice and ideal lattice settings [33]. Subsequently, Tang et al. constructed a new identity-based LRS scheme over NTRU lattice by employing the technologies of trapdoor generation and rejection sampling [34]. In 2022, Cao et al. proposed a new LRS scheme with lattice [35]. In this scheme, ring members’ public keys and the real signer’s private key are both used as the input of a hash function to obtain the output instead of the vector obeying the discrete Gaussian distribution selected in other schemes. In 2023, Cao et al. proposed an efficient LRS based on ideal lattice [36]. Subsequently, in 2024, Zhang et al. designed a lattice-based LRS scheme to provide accurate signature traceability mechanisms for transactions in the blockchain-enabled system [37]. It can protect the system by resisting the quantum attack. Then, Duan et al. presented a novel ring confidential transactions protocol by introducing the linkable threshold ring signature (LTRS) [38]. Afterwards, Xie et al. proposed a linkable, k-times traceable and revocable ring signature (Lk-TRS), which is used to construct a blockchain anonymous transaction system in 2025 [39].

The above method innovatively integrates and utilizes the security features of lattice-based cryptography and LRS, fully leveraging the excellent performance of lattice-based cryptography in resisting quantum computing attacks and the unique advantages of LRS in safeguarding user privacy, thereby achieving significant improvements in both the privacy protection and quantum security of blockchain-enabled IIoT. Unfortunately, in the above LRS schemes, both the amount of signature computation and the size of the signature will increase linearly with the number of ring members. This leads to overall low efficiency and insufficient practicality of the LRS scheme, especially in applications with a large number of users and transactions such as blockchain.

## 3. Lattice-Based LRS Scheme

### 3.1. Formal Definition

Suppose that the number of users in the ring is *k*, and $U=\{ID_{1},ID_{2},\cdots ,ID_{k}\}$ represents all users in the ring.

**Definition 4.**  (Linkable ring signature): The linkable ring signature scheme is usually composed of five PPT algorithms as follows.

***Setup*** ($1^{n}$): Select a security parameter *n*. This Setup algorithm outputs public parameters PP and the master key Mk.

***KeyGen***: Input public parameters, the *KeyGen* algorithm can output the secret key pair (*pk*, *sk*) and a corresponding public key *I*. So, we can obtain a public key set $R=\{pk_{1},pk_{2},\cdots ,pk_{k}\}$.

***Ringsign*** $\left(sk,R,\;M\right)$: Input public key set *R* of the ring, the signer’s key pair (pk,sk) and the message $M\in \{0,1{\}}^{*}$. Run the *Ringsign* algorithm to output signature *e* of message *M*.

***Ringverify*** (*e*, *R*, *M*): Input the public key set *R*, message *M* and ring signature *e*; if the signature e is reasonable, the *Ringverify* algorithm accepts or refuses.

***Link*** $\left(I,v,v^{\prime }\right)$: Take a set $I=\{I_{i}\}$ and two signatures v and $v^{\prime }$. Output “linked” or “independent”.

### 3.2. Security Model

Generally speaking, the linkable ring signature scheme should satisfy three important security properties, namely anonymity, unforgeability and linkability.

**Anonymity**. The anonymity is defined by a game between adversary A with a challenger C as follows.

***KeyGen*** (*n*). Input a security parameter *n*, challenger C runs *KeyGen* algorithm to output a private key *sk* and a public key *pk* for each game participant, and there are *k* participants, $U=\{ID_{1},ID_{2},\cdots ,ID_{k}\}$. So, challenger C obtains the public and private key pairs set $\left\{(pk_{1},sk_{1}),(pk_{2},sk_{2}),\cdots ,(pk_{k},sk_{k})\right\}$.

***Queries.*** Set the public key set $R=\{pk_{1},pk_{2},\cdots ,pk_{k}\}$. Suppose adversary A selects a participant *I* with his public key $pk_{i}\in R$. Challenger C uses participant *I*’s public and private key pair $\left(pk_{i},sk_{i}\right)$ and runs *Ringsign* algorithm to output the signature v; then, he returns v to adversary A.

***Challenge.*** Adversary A submits a message $M^{\prime }$, ring $R^{\prime }$ and two other participants $\{ID_{a_{0}},ID_{a_{1}}\}\in U$. The challenger C chooses a bit b∈{0,1} and runs *Ringsign* algorithm to output signature $v^{\prime }$, then returns $v^{\prime }$ to adversary A.

***Verify.*** If adversary A outputs a guess $b^{\prime }\in \{0,1\}$ and $b^{\prime }=b$, adversary A wins this game.

In this game, suppose that the probability of opponent a winning the game is $Suc_{A}^{anon}(n)$, and the advantage of adversary A is $Adv_{A}^{anon}(n)=Suc_{A}^{anon}(n)-1/2$. If for every probabilistic polynomial-time adversary A, the advantage $Adv_{A}^{anon}(n)$ is negligible, the ring signature scheme is anonymous.

**Unforgeability.** Unforgeability is defined by using the game between an adversary A with a challenger C as follows.

***Setup.*** The challenger C runs *Setup*(*n*) algorithm to generate public parameters *PP* and *MK*, and sends *PP* to the adversary A. Then, adversary A issues *k* queries on identity $U=\{ID_{1},ID_{2},\cdots ,ID_{k}\}$.

***Queries.*** Set the public key set $R=\{pk_{1},pk_{2},\cdots ,pk_{k}\}$. Suppose adversary A selects a participant *I* with his public key $pk_{i}\in R$. Challenger C uses participant *I*’s public and private key pair $\left(pk_{i},sk_{i}\right)$. Adversary A submits a ring $U_{1}\subseteq U$, private key $sk_{i}$ and message *msg*. The challenger C runs *Ringsign* algorithm to output signature v, then returns v to adversary A.

***Forgery.*** The adversary A outputs $\left(U_{2},msg^{\prime },v^{\prime }\right)$, A wins this game if:

(1) *Verify* $\left(PP,U_{2},msg^{\prime },v^{\prime }\right)$ = accept.

(2) Adversary A does not have the private key of the user in $U_{2}$.

(3) $\left(U_{2},msg^{\prime }\right)$ is not submitted to sign query.

**Linkability.** For two different messages $m_{1}$ and ${\;m}_{2}$, the signer can obtain two different signatures $e_{1}$ and $e_{2}$. There exists a PPT algorithm F, which verifies the probability of the same signer is not negligible. On the contrary, if the two different signatures $e_{1}\;$ and $e_{2}\;$ are signed by different signers, the PPT algorithm F verifies that the probability of the same signer is negligible. Thus, the signature scheme is linkable.

### 3.3. Details of Our Scheme

Here, a security parameter n∈Z, a prime q≥2, m≥5nlg⁡q, and $H_{1}:{\left\{0,1\right\}}^{*}\to Z_{q}^{m\times m}$ is a collision-resistant hash function. $H_{2}:Z_{q}^{m}\times \{0,1{\}}^{*}\to \{v=\{-d,\cdots ,d{\}}^{g}:\left\|v\right\|\leq t,d\in N^{+},t\in R\}$. Suppose that there are *k* users in the ring $U=\{ID_{1},ID_{2},\cdots ,ID_{k}\}$. Our linkable ring signature scheme contains the five PPT algorithms as follows.

***Setup* $\left(1^{n}\right)$**: Select and input the security parameter n.

(1) Based on Lemma 2, sender runs $TrapGen(1^{n})$ and obtain a random matrix $A_{0}\in Z_{q}^{n\times m}$ and a corresponding short basis $S_{0}\in {\Lambda_{q}}^{⊥}(A_{0})$. $S_{0}\in Z_{q}^{m\times m}$ is sender’s master key $MK=S_{0}$.

(2) For each user $U=\{ID_{1},ID_{2},\cdots ,ID_{k}\}$, the hash function takes as input *ID*, outputs $R=H_{1}(ID)$ and message $M\in {\left\{0,1\right\}}^{d}$. Thus, the public parameter $PP=\{A_{0},H_{1},H_{2}\}$.

***KeyGen*** (PP,ms,MK): For each member, select each *ID* and input the Gaussian parameter s, *MK*, and *PP*.

Based on Lemma 3, sender runs $BasisDel(A_{0},H_{1}(ID),S_{0},s)$ and obtains his private key $S_{ID}$. Thus, $S_{ID\;}$ is a basis of $\Lambda^{⊥}(A_{0}H_{1}(ID{)}^{-1})$, and his public key $A_{ID}=A_{0}H_{1}(ID{)}^{-1}$.

***RingSign* **$\left(PP,S_{ID_{i}},M,U\right)$: Generate a signature by the following steps.

(1) Randomly select a vector $V\in R^{1\times m}$, compute $I_{i}=VS_{{ID}_{i}}$.

(2) Set $U=\{ID_{1},ID_{2},\cdots ,ID_{k}\}$ and j∈{1,2,⋯,k}, select $s\geq \left\|{\tilde{S}}_{{ID}_{i}}\right\|\omega \left(\sqrt{logm}\right)$, then select vectors $u_{j}\leftarrow D_{s}^{m}$.

(3)
$s_{i}\leftarrow SamplePre\left(A_{{ID}_{i}},S_{{ID}_{i}},s,u\right),x_{i}=s_{i}+u_{i}$

(4) Compute $v=H_{2}(\sum_{j=1}^{k}A_{ID_{j}}u_{j},M)$.

(5) Let j={1,2,⋯,k}, if j≠i, $x_{j}=u_{j}$. If j=i, $x_{j}=x_{i}$.

(6) Output the ring signature $e=(x_{1},x_{2},\cdots ,x_{k},v,I_{i})$.

Verify (PP,U,M,e): Verifier can verify the correctness of this signature e as follows.

(1) For each $x_{j}$ and j∈{1,2,⋯,k}, verify $\left\|x_{j}\right\|\leq s\sqrt{m}$.

(2) Verify $v=H_{2}(\sum_{j=1}^{k}A_{ID_{j}}x_{j}-A_{ID_{i}}s_{i},M)$.

If the above conditions are satisfied, the verifier runs the following *Link* algorithm. Otherwise, this signature *e* will be rejected.

Link (***I***,e): There is a set I that these $I_{i}$ values are stored in, verifier checks if $I_{i}$ has been used in past signatures.

For two signatures $e_{1}=(x_{1},x_{2},\cdots ,x_{k},v_{1},I_{1})$ and $e_{2}=(x_{1},x_{2},\cdots ,x_{k},v_{2},I_{2})$, if $I_{1}=I_{2}$, return 1 (linked) to indicate these two signatures are signed by the same user. If not, return 0 (unlinked).

**Correctness**. Suppose that there is a set l, and j−l={i}, such that

(2) $$\begin{array}{ll} \sum_{j=1}^{k}A_{{ID}_{j}}x_{j}-A_{{ID}_{i}}s_{i} & =\sum_{l=1}^{k}A_{{ID}_{l}}u_{l}+A_{{ID}_{i}}x_{i}-A_{{ID}_{i}}s_{i}\\ & =\sum_{l=1}^{k}A_{{ID}_{l}}u_{l}+A_{{ID}_{i}}\left(s_{i}+u_{l}\right)-A_{{ID}_{i}}s_{i}\\ & =\sum_{l=1}^{k}A_{{ID}_{l}}u_{l}+A_{{ID}_{i}}s_{i}+A_{{ID}_{i}}u_{l}-A_{{ID}_{i}}s_{i}\\ & =\sum_{j=1}^{k}A_{{ID}_{j}}u_{j} \end{array}$$

Such that $v=H_{2}(\sum_{j=1}^{k}A_{ID_{j}}x_{j}-A_{ID_{i}}s_{i},M)$. Therefore, this linkable ring signature scheme satisfies correctness.

## 4. Security Analysis

### 4.1. Anonymity

**Theorem 1.**  *Our proposed ring signature scheme satisfies anonymity.*

**Proof.**  Suppose that there is an adversary A attacking this proposed ring signature scheme based on the anonymity’s definition. □

***KeyGen.*** At first, challenger C selects *k* users to obtain an *ID* set $U=\{ID_{1},ID_{2},\cdots ,ID_{k}\}$. Then, for each user *ID*, input a security parameter *n*; challenger C runs $TrapGen(1^{n})$ algorithm and generates a uniformly random matrix $A_{0}\in Z_{q}^{n\times m}$ with a corresponding short basis $S_{0}\in \Lambda_{q}^{⊥}(A_{0})$. At last, challenger C runs $BasisDel(A_{0},H(ID),S_{0},s)$ to obtain the private key $S_{ID}$. Similarly, a corresponding public key is $A_{ID}=A_{0}H(ID{)}^{-1}$. There are *k* participants $U=\{ID_{1},ID_{2},\cdots ,ID_{k}\}$, and challenger C can obtain the public–private key pairs set $\left\{(pk_{1},sk_{1}),(pk_{2},sk_{2}),\cdots ,(pk_{k},sk_{k})\right\}$.

***Queries.*** Challenger C answers the hash queries, private key queries and signing queries of adversary A. Suppose adversary A selects a participant with his *ID*. Challenger C returns this *ID*’s public and private key pair $\left(pk_{i},sk_{i}\right)$ and runs *Ringsign* algorithm to output signature $e=(x_{1},x_{2},\cdots ,x_{k},v)$, then returns this signature e to adversary A.

***Challenge.*** Adversary A submits a message $M^{\prime }$, ring $U^{\prime }$ and two other participants $\{ID_{a_{0}},ID_{a_{1}}\}\in U$. The challenger C chooses a bit b∈{0,1} and runs *Ringsign* algorithm to output signature $e^{\prime }=(x_{1}^{\prime },x_{2}^{\prime },\cdots ,x_{k}^{\prime },v^{\prime })$, then he returns this signature $e^{\prime }$ to adversary A.

***Guess.*** Adversary A outputs a guess $b^{\prime }\in \{0,1\}$ and verify $b^{\prime }=b$.

For the signature, if $j\neq a_{b}$, $x_{j}=u_{j}$. If $j=a_{b}$, $x_{j}=x_{i}$. According to Lemma 4, $e=(x_{1},x_{2},\cdots ,x_{k},v)$ is not distinguishable from Gauss distribution ${\left(D_{\sigma }^{m}\right)}^{l+1}$. Similarly, $e^{\prime }=(x_{1}^{\prime },x_{2}^{\prime },\cdots ,x_{k}^{\prime },v^{\prime })$ is also not distinguishable from ${\left(D_{\sigma }^{m}\right)}^{l+1}$. We can see that, because these two signatures, *e* and $e^{\prime }$, have the same distribution of the domain, they are computationally indistinguishable.

In summary, under the simulated environment, the adversary A in this anonymity game advantage $Adv_{A}^{anon}(n)=Suc_{A}^{anon}(n)-1/2$ is negligible in guessing the right identity.

### 4.2. Unforgeability

**Theorem 2.**  *Under the lattice SIS problem assumption, the proposed linkable ring signature scheme is existentially unforgeable.*

**Proof.**  Suppose that A is regarded as a PPT adversary. A is able to successfully attack this proposed scheme and forge a new signature. We use ε to denote the probability of success. Then, A is a subroutine, which can solve the lattice short integer solution problem via non-negligible probability. Thus, a PPT algorithm T is constructed, which is realized through interaction with the adversary A as follows. □

**Setup.** The challenger C selects a user set $U=\{ID_{1},ID_{2},\cdots ,ID_{k}\}$ and a user ${ID}_{i}(1\leq i\leq k)$. And C runs the Setup $\left(1^{n}\right)$ algorithm to obtain *PP* and *MK*, and sends *PP* to A. Then, A issues k queries on identity $U=\{ID_{1},ID_{2},\cdots ,ID_{k}\}$.

***KeyGen.*** For 1≤i≤k, challenger C does as follow.

(1) According to Lemma 2, challenger C uses $TrapGen(1^{n})$ to obtain a random matrix $A_{i}\in Z_{q}^{n\times m}$ with a corresponding short basis $S_{i}\in \Lambda^{⊥}(A_{i},q)$. $S_{i}\in Z_{q}^{m\times m}$ is sender’s master key $MK=S_{i}$.

(2) For each user $U=\{ID_{1},ID_{2},\cdots ,ID_{k}\}$, the hash function outputs R=H(ID). For message $M\in {\left\{0,1\right\}}^{d}$. C obtains the public parameter $PP=\{A_{0},H_{1},H_{2}\}$. Then, C transmits *PP* and *U* to A.

(3) Run $BasisDel(A_{i},H(ID_{i}),S_{i},s)$ to generate a secret key $S_{ID_{i}}$ which is a basis of $\Lambda^{⊥}(A_{i}H(ID_{i}{)}^{-1})$. Correspondingly, $A_{ID_{i}}=A_{i}H(ID_{i}{)}^{-1}$.

***Queries.*** Challenger C answers the following hash queries, private key queries and signing queries from adversary A.

(1) ***Hash queries.*** Adversary A chooses a user’s ${ID}_{i}$. Challenger C checks the list $L_{1}$. If adversary A submitted this query before, it will return the same result. Otherwise, Challenger C runs the algorithm $R_{i}=H({ID}_{i})$, returns to adversary A and stores it in $L_{1}$.

(2) ***Private key queries.*** Adversary A selects a user ${ID}_{i}$ from *U*. Challenger C checks the list $L_{1}$ and finds ($A_{i},H(ID_{i}),S_{i},s$). Then, challenger C runs $BasisDel(A_{i},H({ID}_{i}),S_{i},s)$ to return his private key $S_{{ID}_{i}}$ to adversary A and store it in $L_{2}$.

(3) ***Signing queries.*** Adversary A submits a message *M*, a ring $U^{\prime }\subseteq U$ and ${ID}_{i}\in U^{\prime }$. Challenger C runs *Ringsign* algorithm to output ring signature $e=(x_{1},x_{2},\cdots ,x_{k},v)$, then return e to adversary A.

***Forgery.*** Adversary A submits a message $M^{\prime }$, a ring $U_{2}\subseteq U$, a user ${ID}_{a}(1\leq a\leq l)$, adversary A can forge a signature $e^{\prime }=(x_{1}^{\prime },x_{2}^{\prime },\cdots ,x_{l}^{\prime },v^{\prime })$. A wins the game if:

(i) *Verify* $e^{\prime }=(x_{1}^{\prime },x_{2}^{\prime },\cdots ,x_{l}^{\prime },v^{\prime })$ is accepted.

(ii) Adversary A does not have the private key of the user ${ID}_{a}(1\leq a\leq l)$ in $U_{2}$.

(iii) $\left(U_{2},M^{\prime }\right)$ is not submitted to the signing query.

According to our signature scheme, it is shown that if $e^{\prime }=(x_{1}^{\prime },x_{2}^{\prime },\cdots ,x_{l}^{\prime },v^{\prime })$ is a legal signature of ring $U_{2}\subseteq U$, we have

(3) $$A_{{ID}_{j}}{x^{\prime }}_{j}-A_{{ID}_{i}}{s^{\prime }}_{i}=A_{{ID}_{j}}u_{j}$$

Because challenger C can use private key queries to obtain the private key $S_{a}$ of ${ID}_{a}$,${\;e}^{\prime \prime }=(x_{1}^{\prime \prime },x_{2}^{\prime \prime },\cdots ,x_{l}^{\prime \prime },v^{\prime \prime })$ is also a legal signature of ring $U_{2}\subseteq U$, so we have

(4) $$A_{{ID}_{j}}{x^{\prime }}_{j}-A_{{ID}_{i}}{s^{\prime \prime }}_{i}=A_{{ID}_{j}}u_{j}$$

Through the analysis of Equations (3) and (4), we have

(5) $$A_{{ID}_{j}}{x^{\prime }}_{j}-A_{{ID}_{i}}{s^{\prime }}_{i}=A_{{ID}_{j}}u_{j}=A_{{ID}_{j}}{x^{\prime }}_{j}-A_{{ID}_{i}}{s^{\prime \prime }}_{i}$$

So, $A_{{ID}_{i}}{s^{\prime \prime }}_{i}-A_{{ID}_{i}}{s^{\prime }}_{i}=0$; then, we have

(6) $$A_{{ID}_{i}}{(s^{\prime \prime }}_{i}-{s^{\prime }}_{i})=0$$

Let $f=s_{i}^{\prime \prime }-s_{i}^{\prime }$, so $A_{{ID}_{i}}f=0modq$, such that

(7) $$\left\|f\right\|=\left\|s_{i}^{\prime \prime }-s_{i}^{\prime }\right\|\leq \left\|s_{i}^{\prime \prime }\right\|+\left\|s_{i}^{\prime }\right\|\leq \sigma \sqrt{m}+\sigma \sqrt{m}=2\sigma \sqrt{m}$$

Consequently, it means that the result $\left(q,m,2\sigma \sqrt{m}\right)$ is a non-zero solution to the lattice SIS problem.

At last, according to the preimage min-entropy property, the probability of the non-zero solution is not less than $SIS_{s\sqrt{m}}$. The probability that adversary A successfully forges a legal signature is ε, and $pro(i=1)=q_{e}^{-1}$. Therefore, the non-zero solution to the lattice $SIS_{q,m,2\sigma \sqrt{m}}$ problem with a negligible probability $\left(1-2^{-\omega \left(\mathrm{lg}m\right)}\right)q_{e}^{-1}\varepsilon$.

As shown in the calculation and analysis, the probability that adversary A forges a legal and valid signature is negligible. Under the lattice SIS problem assumption, the proposed linkable ring signature scheme satisfies unforgeability. Thus, the proof of this theorem is completed.

### 4.3. Linkability

**Theorem 3.**  *The proposed ring signature scheme is linkable. Formally, adversary A cannot produce n+1 valid signatures $e_{i}$ with key images $I_{i}\neq I_{j}$ for any i,j∈{1,⋯,n}.*

**Proof.**  Suppose that, for the sake of contradiction, adversary A can generate n+1 valid signatures. Since the secret key set $\left|S\right|=n$, there is at least one $I_{i}$ which does not belong to the set **I**. Without a loss of generality, considering this event happened in $e_{x}=(x_{1},x_{2},\cdots ,x_{k},v_{x},I_{x})$, which is a valid signature, we have $I_{x}=VS_{x}$. Because $I_{x}$ does not belong to the set **I**, its secret key $S_{x}$ does not belong to secret key set S. Therefore, $I_{x}\neq I_{i}=VS_{i}$ for i∈{1,⋯,n} and it contradicts previous assumptions, in which adversary A can generate n+1 valid signatures. Consequently, our ring signature scheme is linkable. □

## 5. LRS-Based Blockchain for IIoT

### 5.1. Stealth Address

As described in CryptoNote, the stealth address technology is used in all transactions to provide privacy protection for the receiver. For instance, Alice and Bob have a transaction to make. In this transaction, Alice needs to pay her cryptocurrency to Bob. At first, Alice generates a one-time address for Bob and publishes it as a broadcast in the distributed network. Subsequently, Bob has to check each transaction by using his private key to identify which transaction belongs to him. Subsequently, he recovers this secret key corresponding to the destination address.

Through using a stealth address, the connection of a blockchain transaction’s output with the recipient’s wallet address is broken. More specifically, the actual destination address of a transaction is hidden with the stealth address in CryptoNote. For the sake of protecting the privacy of receivers in blockchain, we also produce stealth addresses as follows, which will be used as the verifying and signing key pairs in the PQB with privacy protection based on lattice.

### 5.2. Blockchain with Privacy Protection

**Figure 1:**
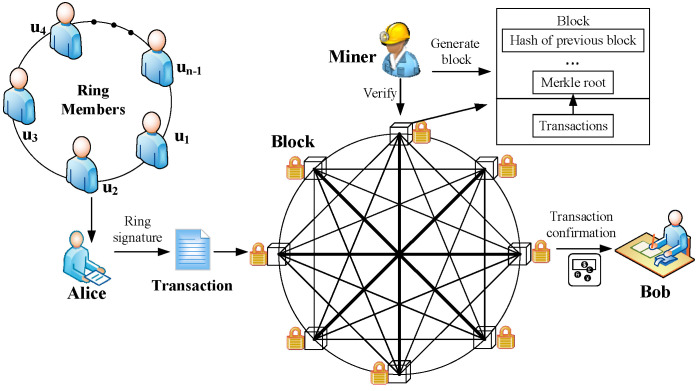
Transaction in PQB with privacy protection.

According to the framework of CryptoNote, in this subsection, we introduce our LRS scheme into blockchain to design a secure PQB scheme with privacy protection for IIoT. Suppose that Alice wants to transfer her cryptocurrency to Bob from her address of a secret key pair $\left(pk_{a},sk_{a}\right)$. As shown in Figure 1, we describe our scheme through the implementation of a transaction as follows.

***Key generation.*** Alice and Bob run the *KeyGen* algorithm to obtain her/his secret key pairs $\left(pk_{a},sk_{a}\right)$ and $\left(pk_{b},sk_{b}\right)$, respectively. It should be noted that Alice’s secret key pair $\left(pk_{a},sk_{a}\right)$ address has been used for receiving the cryptocurrency in the last transaction.

***Key image.*** Bob randomly selects a string b. Then, he calculates hash(b) and sends this hash value to Alice. Then, Alice calculates $Y=E_{pk_{b}}(hash(b))$ and key image $X=hash(sk_{a})$.

**Figure 2:**
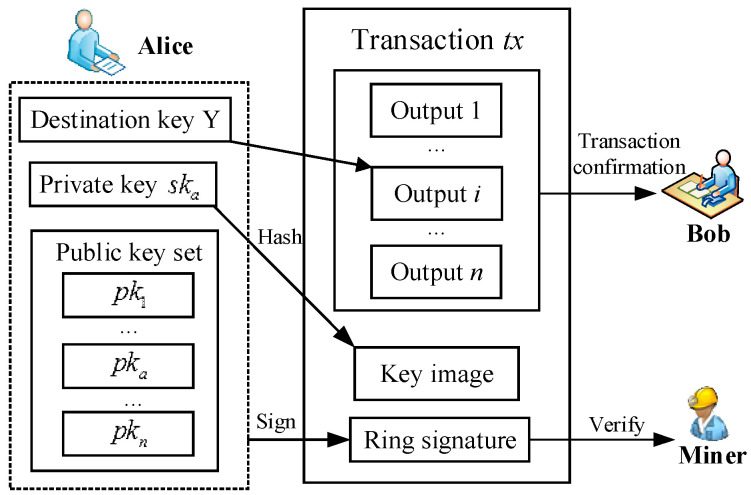
LRS with privacy protection transactions for blockchain.

***Transaction generation.*** Alice specifies *n−1* foreign outputs with the same amount as her outputs and mixes all of these foreign outputs without other people’s participation. All previous transactions with outputs are added to the hash function. Then, this hash value *h* is signed by running the *Ringsign* algorithm, which generates a ring signature e. Afterwards, as shown in Figure 2, she inputs these outputs, *Y*, key image *X*, ring signature *e* and generates a new transaction *tx*.

***Transaction verification.*** By running the *Link* algorithm, the miner nodes verify whether the cryptocurrency in the transaction has been consumed to prevent a double spending attack. Subsequently, miner nodes run the *Verify* algorithm to verify whether the signature of this transaction is correct. If it is correct, this transaction will be encapsulated in a new block. Otherwise, this transaction will be discarded.

***Transaction confirmation.*** Bob checks this transaction. Afterwards, he extracts the destination key from this transaction and calculates $Y^{\prime }=E_{pk_{b}}(hash(b))$. If $Y^{\prime }=Y$, this transaction is the one which Alice sends to Bob. Thus, Bob accepts this transaction, and he records Y with Output i and $\left(pk_{b},sk_{b}\right)$ in his wallet. When he wants to spend this coin with the destination address Y, he can use the corresponding one-time key pair $\left(pk_{b},sk_{b}\right)$ to generate a new transaction as in the above steps.

Different from traditional blockchain, for each ring signature e, it can be checked by using the public key set in our proposed scheme instead of a unique public key. Before the owner uses the same key pair to generate a second signature, the identity of the signer cannot be distinguished from other users in the public key set. More specifically, by using our LRS scheme in PQB for IIoT, the signer’s identity in a ring signature remains anonymous, and two ring signatures, which are signed by the same signer, can be linked. Therefore, this new PQB not only protects the user’s privacy information but also resists the double spending attack. Additionally, as discussed above, the LRS scheme used in PQB satisfies unforgeability. With these above security advantages, the proposed PQB scheme enhances data security for IIoT.

### 5.3. Security and Comparison

As previously highlighted, the lattice-based cryptography algorithm represents a unique mathematical structural model. It has been rigorously demonstrated to possess robust resistance against quantum computing attacks, positioning it as an indispensable component in the future landscape of information security. Consequently, in line with the aforementioned considerations, we opted to employ lattice-based cryptography as the foundational framework for constructing our linkable ring signature scheme. In this study, our linkable ring signature scheme’s security mainly depends on the intractability of the SIS problem from lattice-based cryptography. And the lattice SIS problem in an average case can be reduced to the SIVP in the worst case, which is often used to construct signature schemes for resisting quantum computing attacks [40]. Therefore, our scheme has anti-quantum security.

Furthermore, lattice-based cryptography algorithms are matrix and vector operations, and the computation cost largely determines the cryptography algorithm’s efficiency, especially the public key size and signature size. In this subsection, it is assumed that the parameters (*n, m, q, k*) are the same in our scheme and other related lattice-based ring signature schemes. The detailed comparison results are shown in Table 1. Compared with other schemes, the results show that our public key size and signature size are shorter than those in Refs. [32,33,34,35]. Therefore, our scheme is efficient, with lower computation costs.

Meanwhile, *T_tg_*, *T_bd_*, *T_eb_*, *T_erb_*, *T_rb_*, *T_sp_*, *T_gsp_*, *T_mul_* are set to represent the average consumption time of the following algorithms, TrapGen, BasisDel, ExtBasis, ExtRandBasis, RandBasis, SamplePre, GenSamplePre and vector multiplication, respectively. Then, the master key generation time, user key generation time and signature generation time in these above ring signature schemes are compared, respectively, and the time cost comparison results are shown in Table 2. Among them, our ring signature scheme only uses the TrapGen algorithm once in the master key generation, and the user key generation adopts the BasisDel algorithm k times. Using the rejection sampling lemma, the main steps of generating a ring signature adopt simple vector multiplication. Through this comprehensive comparison, it shows that in the transaction signature process, our scheme’s time cost is less than that in other schemes.

**Figure 3:**
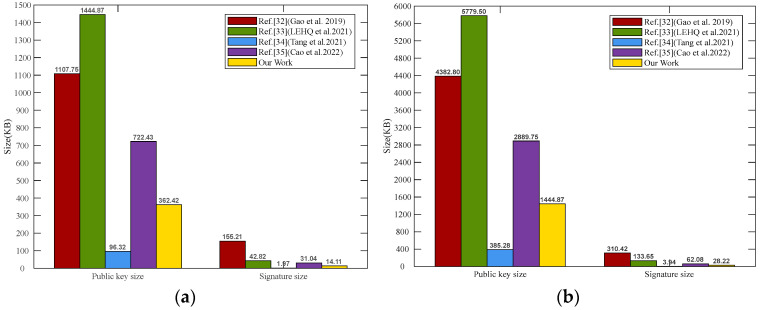
(**a**) Comparison with 80-bit security; (**b**) comparison with 192-bit security [32,33,34,35].

Furthermore, based on the parameters of 80-bit and 192-bit security levels in Ref. [41], and in combination with the parameter requirements of the scheme in our study, the parameters used in the experimental testing are set as follows. We consider two security level of 80-bit and 192-bit, and the parameters polynomial degree n and modulus q are set as n = 256; q = 2^10^, m = 3853 and n = 512; q = 2^10^, m = 7706, respectively. Other reasonable parameters include g = 256, k = 10, $l=\left\lceil \log q\right\rceil =4$. Under two security levels of 80-bit and 192-bit, the public key size, signature size and private key of our proposed scheme and those in Refs. [32,33,34,35] are compared. The simulation results are shown in Figure 3, where (a) and (b) represent the 80-bit security level and 192-bit security level, respectively. As shown in Figure 3, the public key size and signature size of the transaction in our scheme are 362.42 KB and 14.11 KB for 80-bit security, 1444.87 KB and 28.22 KB for 192-bit security. Under the same security levels, our scheme achieves a significant reduction in public key size and signature size compared to Refs. [32,33,35]. In terms of the generated private key size, since the private key sizes in Refs. [32,35] are 4m^2^log*q*, according to the parameter settings of 80-bit and 192-bit security levels, it is obvious that their sizes are much larger than other schemes. Ref. [33]’s generated private key sizes at 80-bit and 192-bit security levels are 458.74 KB and 1834.97 KB, respectively. Ref. [34]’s private key sizes are 0.38 KB and 0.76 KB at 80-bit and 192-bit security levels, respectively. And our scheme’s private key sizes are 0.09 KB for 80-bit security and 0.19 KB for 192-bits security, which are significantly smaller than those in other schemes. After comprehensive comparisons, our LRS scheme for blockchain has lower computational overhead, reduces storage costs, and achieves higher efficiency.

## 6. Conclusions

This study contributes to privacy protection in the process of optimizing user privacy data sharing in blockchain-enabled IIoT. We design an LRS scheme with anti-quantum security and anonymity. This scheme is based on the lattice problem and has superior security. At the same time, the characteristics of LRS can better hide the personal privacy in blockchain. In particular, by combining the stealth address and key image technologies, this scheme is introduced into the construction of blockchain-enabled IIoT, which can support secure data sharing and ensure that data are not tampered with. Meanwhile, the combination of LRS and blockchain-enabled IIoT can effectively enhance the privacy and security of users. Then, it is proved that the LRS scheme in our construction satisfies the security requirements of correctness, unforgeability, anonymity, and linkability. The comparison of key sizes shows that the proposed LRS scheme is efficient and reduces data space overhead. Performance comparisons indicate that our scheme is more practical for IIoT. Our work provides new solutions to the privacy leakage problem of data sharing in the current IIoT system and promotes the application of blockchain in IIoT. In the field of the data sharing of IIoT, there are still several highly promising research directions that urgently need to be explored, especially identity authentication technology, refined data access control mechanisms, and efficient data retrieval strategies. These cutting-edge topics will constitute the content of our future research work.

## Figures and Tables

**Table 1:** Comparison of computation costs.

Scheme	Public Key Size	Private Key	Signature Size
Ref. [32]	(3*m* + *g* + 1)*n*log*q*	4m^2^log*q*	*k*(*k* + 1)*m*log*q*
Ref. [33]	*mnl*log*q*	(*m* + *nl*)*n*log*q*	(*m* + *nl* + *n*)*k* log*q* + *n^2^*log*q* + *n*
Ref. [34]	4*n*^2^log*q*	4*n*log*q*	(2*k* + 1)*n* log*q*
Ref. [35]	2mnlog*q*	4m^2^log*q*	2(k + 1)*m*log*q*
Our work	*mn*log*q*	*n*log*q*	*mk*log*q*

**Table 2:** Comparison of time costs.

Scheme	MK Generation	Secret Key Generation	Signature Generation
Ref. [30]	*T_tg_*	*m T_eb_* + *T_rb_*	*m T_eb_* + *T_rb_* + 2*m T_sp_* + *m*(*k* − *g* + 1) *T_mul_*
Ref. [31]	*k* *T_tg_*	*g* *T_sp_*	*m*(*k* + 1) *T_mul_*
Ref. [32]	*T_tg_*	*k T_erb_*	2*mk T_mul_* + *T_gsp_*
Ref. [33]	*T_tg_*	T_sd_ + T_mul_	(2*k* + 1) *T_mul_*
Ref. [34]	*T_tg_*	*k* T_sp_	(*2k +* 1) *T_mul_*
Ref. [35]	*T_tg_*	*T_rb_* + *m*T_sp_	2(*k* + 1) *T_mul_*
Our work	*T_tg_*	*k* *T_bd_*	*T_sp_* + *mk* Tmul

## Data Availability

Data are contained within the article.
